# Novel approaches to combat chemoresistance against glioblastomas

**DOI:** 10.20517/cdr.2020.38

**Published:** 2020-08-21

**Authors:** Rheal A. Towner, Michelle Zalles, Debra Saunders, Nataliya Smith

**Affiliations:** ^1^Advanced Magnetic Resonance Center, Oklahoma Medical Research Foundation, Oklahoma City, OK 73104, USA.; ^2^Oklahoma Center for Neuroscience, University of Oklahoma Health Sciences Center, Oklahoma City, OK 73104, USA.

**Keywords:** Glioblastoma, pre-clinical models, OKlahoma Nitrone 007, transforming growth factor-β1, ELTD1, magnetic resonance imaging

## Abstract

The poor prognosis of glioblastoma multiforme (GBM) patients is in part due to resistance to current standard-of-care treatments including chemotherapy [predominantly temozolomide (TMZ; Temodar)], radiation therapy and an anti-angiogenic therapy [an antibody against the vascular endothelial growth factor (bevacizumab; Avastin)], resulting in recurrent tumors. Several recurrent GBM tumors are commonly resistant to either TMZ, radiation or bevacizumab, which contributes to the low survival rate for GBM patients. This review will focus on novel targets and therapeutic approaches that are currently being considered to combat GBM chemoresistance. One of these therapeutic options is a small molecule called OKlahoma Nitrone 007 (OKN-007), which was discovered to inhibit the transforming growth factor β1 pathway, reduce TMZ-resistance and enhance TMZ-sensitivity. OKN-007 is currently an investigational new drug in clinical trials for both newly-diagnosed and recurrent GBM patients. Another novel target is ELTD1 (epidermal growth factor, latrophilin and seven transmembrane domain-containing protein 1; alternatively known as ADGRL4, Adhesion G protein-coupled receptor L4), which we used a monoclonal antibody against, where a therapy against it was found to inhibit Notch 1 in a pre-clinical GBM xenograft model. Notch 1 is known to be associated with chemoresistance in GBM. Other potential therapeutic targets to combat GBM chemoresistance include the phosphoinositide 3-kinase pathway, nuclear factor-κB, the hepatocyte/scatter factor (c-MET), the epidermal growth factor receptor, and the tumor microenvironment.

## Introduction

Glioblastoma (GBM) is a devastating primary brain cancer that has a poor prognosis for patients due to limited treatment options. One of the main reasons for poor treatment efficacy is due to chemoresistance. The major chemotherapeutic drug used for GBM is temozolomide (TMZ), and TMZ-resistance is a major reason for tumor recurrence following standard-of-care therapies [surgical resection, radiation therapy, chemotherapy, followed by an anti-angiogenic antibody against vascular endothelial growth factor (VEGF), also known as bevacizumab or Avastin]. The poor efficacy of therapy, and a short interval between remission and recurrence, is thought to be due to the resistance of a small fraction of tumorigenic cells, which are often attributed to cancer stem cells, in their response to treatment^[1]^. There is compelling experimental evidence that suggests that the cancer stem cells present are therapy-resistant glioblastoma stem cells, which subsequently leads to tumor recurrence and subsequent metastasis^[1-5]^. Common gene mutations associated with GBM include, epidermal growth factor receptor (EGFR)^[6]^, IDH1^[7]^, PDGFRA^[8-10]^, HDM2^[11-13]^, PIK3CA^[14,15]^, TERT^[16]^, PIK3R1^[10,15]^, PTEN^[17,18]^, TP53^[19]^, CDKN2A^[20,21]^, NF1^[22]^, ATRX^[23,24]^, and RB^[25]^. Many of these have been investigated regarding therapeutic targets, however efficacy results have been unfruitful in substantially increasing overall survival (OS). There are other genes, proteins and pathways of interest that may provide more promise. The purpose of this review is to identify novel therapeutic approaches to target genes and pathways associated with GBM chemoresistance. In our group, we have developed and characterized two potential therapeutic approaches in pre-clinical GBM xenograft models. One involves a small molecule called OKN-007, which effects the transforming growth factor β1 (TGF-β1) pathway, and is currently in clinical trials for adult GBM. The other is a monoclonal antibody against a novel target, identified by bioinformatics, called ELTD1 or ADGRL4, which is currently being translated for subsequent human trials. In addition to these therapeutic approaches that address chemoresistance in GBM, we will also discuss recent promising therapeutic target developments by other investigators. A summary of the pathways, signaling molecules or tumor environments that can be targeted with therapeutic approaches to decrease tumor drug resistance is presented in Table 1.

**Table 1:** Summary of pathways, signaling molecules, or tumor environments that can be targeted with therapeutic approaches in order to help combat tumor drug resistance

Pathway/target	Therapeutic approaches	Ref.
TGF-β	OKN-007 (TGFβ pathway inhibitor)	[40]
PI3K	BKM120 (PI3K inhibitor)	[55]
	GDC-0941 (PI3K inhibitor)	[53]
NFκB	Parthenolide (NFκB inhibitor)	[61]
c-MET	Endothelial cell-specific knock-out of MET	[63]
Notch	miR-139-5p (oncogene inhibitor)	[64]
	DAPT, MRK-003, GSI-18 (GSIs)	[66,67,71]
	GW280164X, INCB3619 (ASIs)	[72]
	Antibodies against ELTD1	[74,75]
EGFR	Combined therapies targeting EGFR (gefitinib) and mTOR (sirolimus, everolimus)	[82-84]
TME	Targeting macrophages and monocytes	[91]

TGF-β: transforming growth factor β; PI3K: phosphoinositide 3-kinase; NFκB: nuclear factor-κB; EGFR: epidermal growth factor receptor; TME: tumor microenvironment; GSIs: γ-secretase inhibitors; ASIs: α-secretase inhibitors

## Therapeutic options for TMZ-chemoresistance in GBM

### OKN-007 targeting of the TGF-β1 pathway

TGF-β signaling drives the regulation of proliferation, differentiation and survival/or apoptosis of several cells, including glioma cells^[26]^. TGF-β acts through explicit receptors that activate multiple intracellular pathways, resulting in the phosphorylation of receptor-regulated Smad2/3 proteins that are associated with the common mediator, Smad4^[26-28]^. This complex translocates to the nucleus, and subsequently binds to DNA and regulates the transcription of several genes^[26]^. In addition, TGF-β-activated kinase-1 is an element of TGF-β signaling, and activates mitogen-activated protein kinase (MAPK) cascades^[26]^. Negative regulation of TGF-β/Smad signaling often occurs through the inhibitory Smad6/7 signaling path^[26,29,30]^. Although the genetic alterations in TGF-β genes related to signaling are relatively infrequent in gliomas, the altered expression of those genes is a frequent event^[26]^. The increased expression of TGF-β1-3 correlates with the degree of malignancy in human gliomas^[26,31]^. TGF-β may contribute to tumor pathogenesis in several means, such as, via the direct support of tumor growth^[26,32]^, via maintaining self-renewal of glioma initiating stem cells^[26,33,34]^, and by inhibiting anti-tumor immunity^[26,35]^. Glioma initiating cells are thought to be dedifferentiated cells that maintain many stem cell-like properties, and play a role in tumor initiation, as well as contributing to tumor recurrence^[26]^. TGF-β1,2 stimulates the expression of VEGF, the plasminogen activator inhibitor, and some metalloproteinases that are implicated in vascular remodeling, angiogenesis and degradation of the extracellular matrix^[26,36-38]^. Inhibitors of TGF-β signaling have been found to reduce the proliferation and subsequent invasion of gliomas in animal models, and could provide a path forward for developing promising anti-tumor therapeutics^[39,40]^.

There is a differential expression of TGF-β1 in GBM tumors^[41]^. Specifically, it has been reported that there was a significant relationship between TGF-β1 expression and OS and progression free survival in newly diagnosed GBM^[41]^. It has been found that dysregulated TGF-β signaling leads to a cascade of events that contribute to oncogenesis^[42,43]^, which includes decreased apoptosis^[42,44]^, up-regulated proliferation^[42,45]^, immune surveillance evasion^[42,46]^, and an epithelial-to-mesenchymal transition (EMT)^[42,47]^.

**Figure 1:**
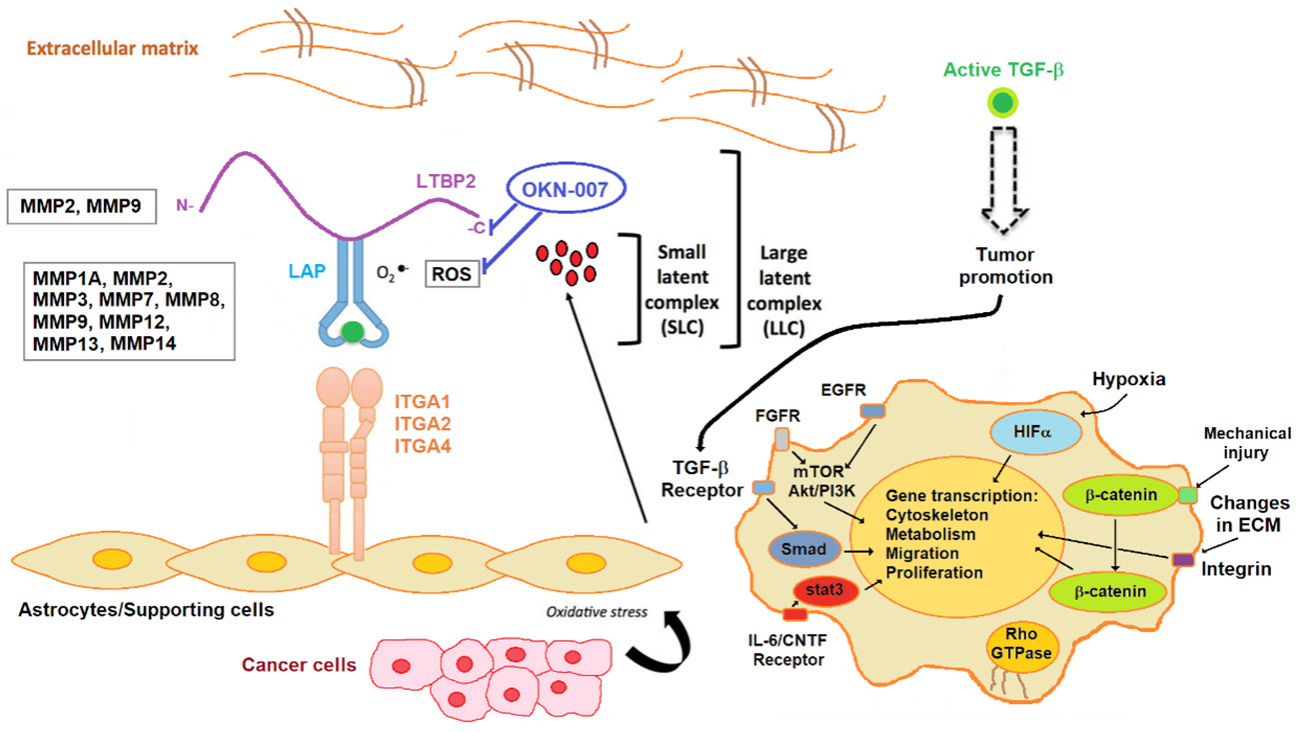
Stromal activators of transforming growth factor-β (TGF-β) in the tumor microenvironment. MMP2 and MMP9 proteolytically cleave latent TGF-β binding protein (LTBP), thereby releasing latent TGF-β from the extracellular matrix. MMP1A, MMP2, MMP3, MMp7, MMP8, MMP9, MMP12, MMP13 and MMP14 activate latent TGF-β via proteolytic cleavage of the latency-associated peptide (LAP), while integrins expressed on astrocytes (ITGA1, 2 and 4) bind to the large latent complex (LLC) and activate latent TGF-β through MMP-dependent cleavage of LAP. Integrins (ITGA1, 2 and 4) bind to the LLC and induce conformational changes in the latent complex via contractile action from activated astrocytes. Reactive oxygen species (ROS) produced by activated astrocytes via the induction of oxidative stress from adjacent cancer cells can lead to the oxidation of the LAP domain and induce allosteric changes that release mature TGF-β from LAP. The mature (active) form of TGF-β can then bind to its cognate receptor and exert its tumor promoting and tumor suppressive properties. Dashed arrow indicates recruitment of the mature TGF-β protein to its cognate receptor. Other tumor-associated pathways/signaling molecules include fibroblast growth factor receptor (FGFR), EGFR, mammalian target of rapamycin (mTOR)/Akt/PI3K, HIFα (hypoxia inducible factor α), β-catenin, and stat3 (signal transducer and activator of transcription 3) (via the IL-6/CNTF receptor). Modified from Costanza *et al*.^[104]^ (2017). Based on microarray and RT-PCR data from the rat F98 glioma model, comparing untreated to OKN-007-treated tumor tissue, OKN-007 is thought to act on LTBP^[40]^, as well as ROS^[105]^. LTBP2, MMP1A, MMP2, MMP3, MMP7, MMP8, MMP9, MMP12, MMP13 and MMP14, were all found to be downregulated in microarray and/or RT-PCR data from the F98 glioma study^[40]^. Modified with permission from Dr. Towner, which was originally published in Towner *et al*.^[40]^ (2019)

**Figure 2:**
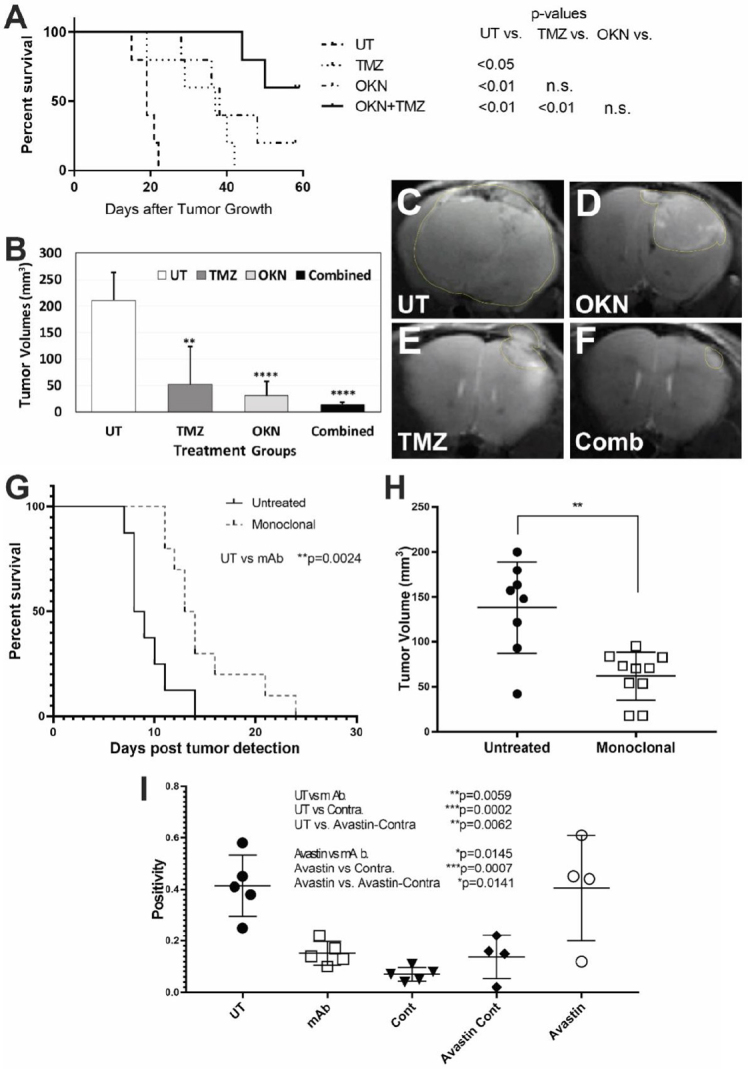
Targeting either the transforming growth factor β1 pathway or ELTD1 in pre-clinical studies for glioblastoma multiforme (GBM). OKlahoma Nitrone 007 (OKN-007) is able to significantly increase animal survival (A) or decrease tumor volumes (B) following combined OKN-007 and TMZ treatments in an orthotopic G55 GBM xenograft model. Examples of MR images from untreated (UT) (C), OKN-007- (OKN) (D), TMZ- (E), or combined (OKN-007 + TMZ) (F) treatments; a monoclonal antibody (mAb) against ELTD1 significantly increased animal survival (G) and decreased tumor volumes (H) in an orthotopic G55 GBM xenograft model; (I) Notch 1 levels were significantly decreased with a mAb against ELTD1 in a G55 GBM model. This figure was obtained from modified data with the permission of Dr. Towner, as reported in Towner *et al*.^[40]^ and Zalles *et al*.^[74]^

We have previously found that OKN-007 increases TMZ sensitivity and also suppresses TMZ-resistant GBM tumor growth^[40]^. OKN-007 seems to elicit its effect on GBM tumors by inhibiting tumorigenic TGF-β1, mainly by affecting the extracellular matrix [Figure 1]^[40]^. When combined with TMZ, OKN-007 was found to significantly increase percent survival [Figure 2A], decrease tumor volumes [Figure 2B-F], and normalize tumor blood vasculature *in vivo* compared to untreated tumors in a human GBM G55 orthotopic xenograft model^[40]^. It is known that TGF-β1 plays a major role in TMZ-resistance^[48,49]^, and we believe that OKN-007 may actually be affecting TMZ-resistance by targeting TGF-β1.

When we obtained RNA-seq data for TMZ-resistant LN18 human GBM cells, and compared the combined TMZ + OKN treatment group to TMZ-treatment alone, we found an interesting downregulated gene, SUMO2^[48]^, that seems to be directly associated with treatment resistance. It was previously found that overexpression of SUMO, which occurs in conditions such as brain ischemia and hypoxia, could increase cell survival, whereas in contrast, the knockdown of SUMO expression has been shown to be toxic to cells and is associated with TGFβ1 in resistant glioma cells^[48]^. In particular, SUMOylation has been found to regulate TGF-β1/Smad4 signaling in-resistant glioma cells^[49]^.

### Targeting the phosphoinositide 3-kinase pathway

Phosphatidylinositol-3 kinases, PI3Ks, comprise of a lipid kinase family that are characterized by their ability to phosphorylate the inositol ring 3’-OH group in inositol phospholipids, which leads to the generation of a second messenger phosphatidylinositol-3,4,5-trisphosphate (PI-3,4,5-P 3)^[50,51]^. Subsequently, receptor protein tyrosine kinase activation results in PI (3,4,5)P 3 and PI (3,4)P 2 production by PI3K at the inner side of the cellular plasma membrane^[51,52]^. protein kinase B (Akt) then interacts with these phospholipids, resulting in its translocation to the inner membrane, where it then becomes phosphorylated and activated by PDK1 and PDK2^[51,52]^. Activated Akt is known to modulate the function of numerous substrates that are involved in the regulation of cellular growth, cell cycle progression, and cell survival^[51]^. More recently it has been shown that several components of the PI3K/Akt signaling pathway are commonly altered in human cancers^[51]^. It is widely known that cancer treatments by chemotherapy and γ-irradiation kills target cells primarily by the induction of apoptosis^[51]^. Unfortunately, resistance to therapy commonly occurs, and is a major clinical problem that needs to be solved. Failure to activate apoptosis is characteristic as an important mechanism of drug resistance in tumor cells^[51]^. As cell survival signals are known to be induced by several receptors mediated by PI3K/Akt, it is anticipated that this pathway may substantially contribute to the generation of resistant phenotypes^[51]^.

It has also been established that GBM is also characterized by overt activity of the PI3K signaling pathway^[53]^. The activity of the PI3K-Akt signaling pathway is correlated with higher cell survival and motility, as well as chemotherapeutic resistance^[53]^. Inhibition of the PI3K pathway has been shown to sensitize human glioma cells to alkylating drugs^[54]^. For instance, PI3K inhibitors such as BKM120 have revealed decreased proliferation and increased apoptosis in not only tumor cell lines^[55]^, and tumor xenograft models^[55]^, but also cancer patients with PI3K activating mutations^[55]^.

It has been previously shown that following TMZ treatment and within TMZ resistant GBM biopsies, there was a distinct activation pattern of the PI3K signaling cascade, further indicating that this pathway is involved in chemoresistance^[54]^. This pathway was also found to be activated in GBM cell lines^[54]^. The PI3K pathway seems to play a crucial role in resistance to alkylating agents, and should therefore be considered as a potential drug target for chemosensitization^[54]^. As an example, the highly specific PI3K inhibitor GDC-0941, was found to reduce chemoresistance to TMZ and enhance radiosensitization in GBM cell lines^[53]^.

Microarray data from OKN-007-treated F98 glioma-bearing rats indicated that OKN-007 also inhibited the PI3K pathway^[40]^, further supporting the use of this investigative drug as a therapeutic option to combatting TMZ-chemoresistance.

### Targeting nuclear factor κB

The Nuclear factor kappa-light-chain-enhancer of activated B cells (NF-κB) comprises of a family of 5 transcription factors, NF-κB1/p50 (p50), NF-κB2/p52 (p52), Rel-like domain containing protein A (RelA), RelB, and c-Rel, that form either heterodimers or homodimers, and subsequently bind onto DNA sequences to regulate cellular processes such as apoptosis, DNA repair, innate immunity, and cell proliferation^[56,57]^.

Of importance, NF-κB is increasingly recognized as a critical participant in many steps of cancer initiation and progression^[56]^. During these processes NF-κB works together with multiple other signaling molecules and pathways^[56]^. NF-κB is activated via different stimuli, which include growth factors and reactive oxygen species, as well as DNA damage and oncogenic stress from cells^[57,58]^. Nodes of crosstalk signaling are also mediated by other transcription factors such as p53 and STAT3, or also the ETS (erythroblast transformation-specific) related gene. It is thought that these transcription factors either directly interact with NF-κB subunits or affect NF-κB target genes^[56]^. It is also known that crosstalk can occur via different kinases, such as PI3K, GSK3-β, or p38, which subsequently modulate NF-κB transcriptional activity or can be involved in upstream signaling pathways^[56]^. Other types of molecules that act as nodes of crosstalk include miRNAs and reactive oxygen species^[56]^.

Increased NF-κB activity in GBM has been correlated with poor prognosis and enhanced risk resistance to radiation and chemotherapy by promoting tumor initiation and progression, involving the stimulation of cell proliferation, tumor angiogenesis, and prohibition of apoptosis^[58,59]^. Studies with GBM stem-like cells referred to as tumourspheres showed that inhibiting endogenous NF-κB activity either via selective antagonist of inhibitors of the κB (IκB) kinase (IKK) complex (IKKβ) or siRNA knockdown, which are involved in playing key roles in the activation of the canonical pathway of NF-κB, resulted in decreased tumorspheres formation^[60]^. NF-κB activation promotes the maintenance of GBM-stem-like cells therefore suggesting an impact on GBM resistance to radiotherapy by increasing the percentage of stem-like behavior in cancer cells^[60]^.

It has been shown that inhibition of NF-κB results in anti-glioma activity, and also reduces TMZ-induced chemoresistance via down-regulation of O6-methylguanine-DNA methyltransferase (MGMT) gene expression^[61]^. More specifically, treating various established glioma cell lines with pharmacological NF-κB inhibitors resulted in markedly decreasing glioma viability, also leading to S cell cycle arrest, as well as inducing apoptosis^[61]^. It has been established that there is a signiﬁcant correlation between NF-κB expression and MGMT expression in gliomas from different origins, as confirmed with immunohistochemistry assessments^[61]^. As an example, parthenolide inhibition of NF-κB activity was found to down-regulate MGMT gene expression, and this resulted in substantially restoring TMZ chemosensitivity as assessed in both *in vitro* and *in vivo* experiments^[61]^.

### Targeting the hepatocyte/scatter factor

There is increasing evidence that suggests the expression levels of the receptor tyrosine kinase c-MET, and its stimulatory factors, are found to be significantly increased in GBM, in comparison to normal brain tissues, whereas many of the negative regulators are conversely downregulated^[62]^. It has been found that mutations in c-MET, as well as the dysregulation of other regulators of crosstalk associated with c-MET signaling pathways, are also characteristic in GBM^[62]^. c-MET and its ligand hepatocyte growth factor, or the scatter factor, play critical roles in survival, proliferation, invasion, migration, angiogenesis, promotion of stem cell characteristics, as well as therapeutic resistance and recurrence in GBM^[62]^. Combined targeted therapies for c-MET and associated signaling molecules could provide beneficial strategies for the potential treatment of human GBM.

It has also been established that c-MET-mediated endothelial plasticity is known to drive aberrant vascularization and chemoresistance in GBM^[63]^. Endothelial cell-specific knock-out of MET was found to inhibit vascular transformation, result in the normalization of blood vessels, reduce intratumoral hypoxia, leading to suppressed tumor growth and prolonged survival in GBM-bearing mice following TMZ treatment^[63]^. Taken together, these findings suggest that targeting the EMT may offer selective and beneficial strategies that can result in anti-vascular and vessel normalization therapies in GBM^[63]^.

### Targeting Notch

The Notch signaling pathways are highly evolutionarily conserved, and are crucial for cell differentiation, proliferation, migration, and tumor angiogenesis^[64-66]^. Notch homologous proteins- Notch1, Notch2, Notch3, and Notch4- are 300-kD single-pass transmembrane proteins that include a Notch intracellular domain (NICD) and an extracellular domain (NECD)^[66]^. The Notch pathway is activated via the binding of one of the ligand families, Delta-like (Dll-3 and Dll-4) and Jagged (Jagged-1 and -2), to the NECD via direct cell-to-cell contact^[66]^. Once bound, the Notch receptor undergoes a conformational change that enables a member of the ADAM-family to cleave the NECD^[66]^. This cleavage releases the NECD-ligand complex, that is then taken up by the signal-sending cell via endocytosis, resulting in the expression of Delta-like or Jagged ligands on its cell membrane surface^[66,67]^. The γ-secretase complex then cleaves the Notch receptor of the signal-receiving cell, resulting in the expression of the Notch receptor on its cell membrane surface, releasing NICD and allowing it to translocate to the nucleus to activate the expression of target genes^[66,67]^.

Deregulation of the Notch pathways results in a variety of diseases including various cancers. In GBM, Notch1 overexpression is correlated with low OS as well as increased expression of VEGF^[65]^. There is accumulating evidence that suggests that Notch1 plays an important role in tumor progression^[65,66]^. Notch1 was found to be upregulated in glioma tissues and cell lines, and positively associated with increasing tumor grade^[64,65]^. Additionally, Notch1 upregulation was positively correlated with stem cell markers such as CD133 and Nestin^[68,69]^. Knockdown (KD) of the Notch pathway, via KD of the Notch1 gene, have demonstrated decreased Notch receptor expression, along with decreased proliferation and formation of cancer stem cells and increased apoptosis^[66]^. MicroRNAs (miRNAs or miRs) are a class of small non-coding RNAs whose role is to regulate gene expression after transcription. Targeting the Notch oncogene with miR-139-5p inhibits glioma metastasis and EMT^[64]^. In GBM there is a downregulation of miR-34a, which directly targets Notch1, compared to normal brain tissue^[70]^. An overexpression of miR-34a led to a suppression of proliferation, as well as an increase of glioblastoma cellular apoptosis^[70]^.

A complete shutdown of the Notch signaling pathway is not required in order to have a therapeutic effect. Instead, some groups have focused on γ-secretase inhibitors (GSIs) or α-secretase inhibitors (ASIs). Notch signaling pathway activation heavily relies on the γ-secretase complex to cleave the active NICD from the Notch receptor^[65]^. One of the most well-known GSIs is DAPT (GSI-IX) which has been shown to reduce glioma stem cell proliferation and induce apoptosis mediated by a reduction of NF-κB^[67,71]^. DAPT, as well as other GSIs such as MRK-003 and GSI-18, have shown decreased cancer stem cells, decreased invasion, increased survival in animal models, and most importantly, have enhanced TMZ and radiation treatments^[66]^. ASIs target ADAM10 and ADAM17, surface proteins that cleave the NECD^[66]^. ASIs such as GW280164X and INCB3619 had decreased Notch activity, decreased cancer stem cells, and decreased cell growth^[72]^.

Notch signaling also has an effect on tumor microvasculature by regulating the sprouting process^[73]^. In endothelial cells, VEGF-A binds onto and activates VEGF-receptor 2 (VEGFR2) in the tip cell^[73]^. This activation leads to the activation of the DLL-4 promoter that has two main responsibilities, to increase the levels of VEGFR2 in the tip cells as well as to activate the Notch receptor in the neighboring cell^[73]^. In normal vasculature, Notch receptor activation causes transcriptional activation of Hes (Hairy/Enhancer of split) family members that repress the VEGFR2 promotor in the stalk cell^[73]^. This regulatory process prevents abnormal sprouting from occurring in the normal healthy brain^[67]^. However, in GBM this process is destabilized allowing for high VEGF and Notch levels resulting in unregulated angiogenesis, the formation of new blood vessels from pre-existing vessels^[67]^. Targeting DLL-4, either via ionizing radiation or deletion of the gene encoding DLL-4, has shown to slow the progression of GBM cancer cell lines by decreasing angiogenesis^[67]^.

ELTD1 is a novel regulator of brain angiogenesis that is highly expressed in human high-grade gliomas^[74]^. In normal vasculature, VEGF increases ELTD1 expression while Notch/DLL-4 signaling decreases the expression^[74,75]^. Antibodies against ELTD1 showed a normalization of the vasculature, along with increased overall animal survival [Figure 2G for mAb], and decreased tumor volumes [Figure 2H for mAb]^[74,76]^. Furthermore, targeting ELTD1 with polyclonal (pAb), monoclonal (mAb), or single-chain variable fragment region (scFv) antibodies, showed a drastic decrease of Notch1 expression levels in a GBM mouse model [Figure 2I for mAb]^[74,76]^. Additionally, RNA-sequencing data has shown that targeting/silencing of ELTD1 affects Notch pathway genes and also down-regulates Nestin-related pathways^[74,77]^.

### EGFR drug resistance

It is well established that the epidermal growth factor receptor is overexpressed^[78]^, mutated and amplified in high-grade gliomas^[78-81]^. Resistance to EGFR-targeted therapies in high-grade gliomas has been established to occur via both EGFR-dependent and EGFR-independent mechanisms^[81]^. For instance, via the use of combined genetic and pharmacologic interventions (e.g., gefitinib, erlotinib, lapatinib and cetuximab), it was discovered that EGFR-associated gliomas were not responsive to EGFR tyrosine kinase inhibitors, but could inhibit EGFR-related autophosphorylation^[81]^. It was also established that even though genetic suppression of EGFR was found to initially lead to tumor regression and increased animal survival, all tumors eventually recurred and had increased tumor growth^[81]^. There is some evidence that implies that PTEN plays an important role in predicting GBM response to EGFR-targeted therapy^[82]^. An aberrant Akt/mammalian target of rapamycin (mTOR) pathway has also been shown to contribute to the resistant phenotype. Gefitinib and mTOR inhibitors (sirolimus^[83]^, and everolimus^[84]^) have been found to improve individual drug resistance^[82]^. Multiple-target therapies may provide more promising approaches to circumvent EGFR drug resistance.

### Targeting the tumor microenvironment

The tumor microenvironment (TME) includes non-cancerous cells, found within and surrounding the tumor, including for instance immune cells, microglia/macrophages, and astrocytes^[85]^. Additionally, the TME also includes the proteins and non-protein biomolecules that are produced by the cell types that help with tumor progression^[86]^. Some of the major pathways that contribute to the TME include PIK3 and its associated immune checkpoint ligand PD-1^[87]^, Ras-MAPK, which is down-stream of EGFR, via the induction of IL6^[88]^, TGFβ^[89]^, and WNT/β-catenin^[90,91]^. It has been shown from recent studies that the TME plays a critical role in the chemoresistance of several different tumors, which suggests that some of its components could be suitable targets for various cancer therapies, and can also be important for prognostic purposes^[92]^.

Regarding GBM, the TME plays a fundamental role to regulate tumor progression, and also contributes to therapeutic resistance, particularly as it is highly immunosuppressive^[93-96]^. The TME comprises of numerous types of stromal, endothelial and immune cells^[97]^. These cells are recruited by cancer stem cells (CSCs), and they tend to influence CSC phenotypes and their respective behaviors^[97]^. The TME also promotes acidosis and hypoxia, both of which have been shown to play critical roles in GBM chemoresistance, resulting in the interference associated with apoptosis, angiogenesis, DNA repair, oxidative stress, immune escape, and the expression and activity of multi-drug resistance-related genes^[97]^. The blood brain barrier (BBB), which protects the brain microenvironment from blood, ends up being a major barrier for the delivery of chemotherapy agents into the brain, and also plays a role in drug-resistant phenotypes of GBM^[97]^.

Astrocytes are involved in playing an important role for the BBB, and the tripartite synapse neural network, which helps promote bidirectional communication with neurons under physiological conditions^[93]^. There is emerging evidence that shows that tumor-associated reactive astrocytes interact with glioma cells and facilitate the aggression, progression, and survival of tumors by releasing different cytokines^[93,98,99]^. The communication between reactive astrocytes and glioma cells is further promoted via ion channels and ion transporters, which increase the migratory capacity and invasiveness of tumor cells via changing H^+^ and Ca^2+^ concentrations, and therefore stimulating volume changes in cells^[93,100-103]^. This subsequently contributes to the loss of epithelial polarization, resulting in the initiation of the EMT^[92]^.

As macrophages and monocytes, which have protumor and immunosuppressive effects, make up the majority of infiltrating immune cells, targeting these cells may influence the GBM TME, and provide a promising therapeutic approach^[91]^.

## Conclusion

There are several gene or protein targets that are currently being investigated either pre-clinically or clinically as possible solutions to overcome GBM chemoresistance. These include the TGF-β1 pathway, the PI3K pathway, NF-κB, c-MET, Notch-1, and the TME. Promising pre-clinical or clinical research was discussed by targeting these pathways and/or gene/protein targets that may contribute to the arsenal of therapeutic approaches that can combat GBM chemoresistance. Two promising therapeutic approaches being investigated by our research group include the small molecule OKN-007 and an antibody therapy against ELTD1.

## Declarations

### Availability of data and materials

Available through published results, and upon request.

### Copyright

© The Author(s) 2020.

## References

[1] Sharifzad F, Ghavami S, Verdi J, Mardpour S, Mollapour Sisakht M (2019). Glioblastoma cancer stem cell biology: potential theranostic targets.. Drug Resist Updat.

[2] Akbari-Birgani S, Paranjothy T, Zuse A, Janikowski T, Cieślar-Pobuda A (2016). Cancer stem cells, cancer-initiating cells and methods for their detection.. Drug Discov Today.

[3] Farahani E, Patra HK, Jangamreddy JR, Rashedi I, Kawalec M (2014). Cell adhesion molecules and their relation to (cancer) cell stemness.. Carcinogenesis.

[4] Hombach-Klonisch S, Mehrpour M, Shojaei S, Harlos C, Pitz M (2018). Glioblastoma and chemoresistance to alkylating agents: involvement of apoptosis, autophagy, and unfolded protein response.. Pharmacol Ther.

[5] Wasik AM, Grabarek J, Pantovic A, Cieślar-Pobuda A, Asgari HR (2014). Reprogramming and carcinogenesis-parallels and distinctions.. Int Rev Cell Mol Biol.

[6] Mazzoleni S, Politi LS, Pala M, Cominelli M, Franzin A (2010). Epidermal growth factor receptor expression identifies functionally and molecularly distinct tumor-initiating cells in human glioblastoma multiforme and is required for gliomagenesis.. Cancer Res.

[7] Cohen AL, Holmen SL, Colman H (2013). IDH1 and IDH2 mutations in gliomas.. Curr Neurol Neurosci Rep.

[8] Furnari FB, Cloughesy TF, Cavenee WK, Mischel PS (2015). Heterogeneity of epidermal growth factor receptor signalling networks in glioblastoma.. Nat Rev Cancer.

[9] Koschmann C, Zamler D, MacKay A, Robinson D, Wu YM (2016). Characterizing and targeting PDGFRA alterations in pediatric high-grade glioma.. Oncotarget.

[10] Cancer Genome Atlas Research Network (2008). Comprehensive genomic characterization defines human glioblastoma genes and core pathways.. Nature.

[11] de Toledo SM, Azzam EI, Dahlberg WK, Gooding TB, Little JB (2000). ATM complexes with HDM2 and promotes its rapid phosphorylation in a p53-independent manner in normal and tumor human cells exposed to ionizing radiation.. Oncogene.

[12] Lathia JD, Liu H (2017). Overview of cancer stem cells and stemness for community oncologists.. Target Oncol.

[13] Noushmehr H, Weisenberger DJ, Diefes K, Phillips HS, Pujara K, Cancer Genome Atlas Research Network (2010). Identification of a CpG island methylator phenotype that defines a distinct subgroup of glioma.. Cancer Cell.

[14] Gallia GL, Rand V, Siu IM, Eberhart CG, James CD (2006). PIK3CA gene mutations in pediatric and adult glioblastoma multiforme.. Mol Cancer Res.

[15] Zhao HF, Wang J, Shao W, Wu CP, Chen ZP (2017). Recent advances in the use of PI3K inhibitors for glioblastoma multiforme: current preclinical and clinical development.. Mol Cancer.

[16] Beck S, Jin X, Sohn YW, Kim JK, Kim SH (2011). Telomerase activity-independent function of TERT allows glioma cells to attain cancer stem cell characteristics by inducing EGFR expression.. Mol Cells.

[17] Benitez JA, Ma J, D’antonio M, Boyer A, Camargo MF (2017). PTEN regulates glioblastoma oncogenesis through chromatin-associated complexes of DAXX and histone H3.3.. Nat Commun.

[18] Zheng H, Ying H, Yan H, Kimmelman AC, Hiller DJ (2008). p53 and Pten control neural and glioma stem/progenitor cell renewal and differentiation.. Nature.

[19] Daniele S, Taliani S, Da Pozzo E, Giacomelli C, Costa B (2014). Apoptosis therapy in cancer: the first single-molecule co-activating p53 and the translocator protein in glioblastoma.. Sci Rep.

[20] Kanamori M, Suzuki H, Takei H, Sonoda Y, Uenohara H (2016). Malignant transformation of diffuse astrocytoma to glioblastoma associated with newly developed BRAF V600E mutation.. Brain Tumor Pathol.

[21] Parsons DW, Jones S, Zhang X, Lin JC, Leary RJ (2008). An integrated genomic analysis of human glioblastoma multiforme.. Science.

[22] Verhaak RG, Hoadley KA, Purdom E, Wang V, Qi Y, Cancer Genome Atlas Research Network (2010). Integrated genomic analysis identifies clinically relevant subtypes of glioblastoma characterized by abnormalities in PDGFRA, IDH1, EGFR, and NF1.. Cancer Cell.

[23] Appin CL, Brat DJ (2014). Molecular genetics of gliomas.. Cancer J.

[24] Schwartzentruber J, Korshunov A, Liu XY, Jones DT, Pfaff E (2012). Driver mutations in histone H3.3 and chromatin remodelling genes in paediatric glioblastoma.. Nature.

[25] Cenciarelli C, Marei HE, Felsani A, Casalbore P, Sica G (2017). Correction: PDGFRα depletion attenuates glioblastoma stem cells features by modulation of STAT3, RB1 and multiple oncogenic signals.. Oncotarget.

[26] Kaminska B, Cyranowski S, Barańska J (2020). Recent advances in understanding mechanisms of TGFβ signaling and its role in glioma pathogenesis.. Glioma signaling..

[27] Brown KA, Pietenpol JA, Moses HL (2007). A tale of two proteins: differential roles and regulation of Smad2 and Smad3 in TGF-β signaling.. J Cell Biochem.

[28] Budi EH, Duan D, Derynck R (2017). Transforming growth factor-β receptors and Smads: regulatory complexity and functional versatility.. Trends Cell Biol.

[29] Derynck R, Zhang YE (2003). Smad-dependent and Smad-independent pathways in TGF-β family signalling.. Nature.

[30] Itoh S, ten Dijke P (2007). Negative regulation of TGF-β receptor/Smad signal transduction.. Curr Opin Cell Biol.

[31] Hau P, Jachimczak P, Schlaier J, Bogdahn U (2011). TGF-β2 signaling in high-grade gliomas.. Curr Pharm Biotechnol.

[32] Bruna A, Darken RS, Rojo F, Ocaña A, Peñuelas S (2007). High TGFβ-Smad activity confers poor prognosis in glioma patients and promotes cell proliferation depending on the methylation of the PDGF-B gene.. Cancer Cell.

[33] Ikushima H, Todo T, Ino Y, Takahashi M, Miyazawa K (2009). Autocrine TGF-β signaling maintains tumorigenicity of glioma-initiating cells through Sry-related HMG-box factors.. Cell Stem Cell.

[34] Peñuelas S, Anido J, Prieto-Sánchez RM, Folch G, Barba I (2009). TGF-β increases glioma-initiating cell self-renewal through the induction of LIF in human glioblastoma.. Cancer Cell.

[35] Beck C, Schreiber H, Rowley DA (2001). Role of TGF-β in immune-evasion of cancer.. Microsc Res Tech.

[36] Sánchez-Elsner T, Botella LM, Velasco B, Corbí A, Attisano L (2001). Synergistic cooperation between hypoxia and transforming growth factor-β pathways on human vascular endothelial growth factor gene expression.. J Biol Chem.

[37] Wick W, Platten M, Weller M (2001). Glioma cell invasion: regulation of metalloproteinase activity by TGF-β.. J Neurooncol.

[38] Dietrich LC, Mellberg S, Langenkamp E, Zhang L, Zieba A (2012). Transcriptional profiling of human glioblastoma vessels indicates a key role of VEGF-A and TGFβ2 in vascular abnormalization.. J Pathol.

[39] Vogt J, Traynor R, Sapkota GP (2011). The specificities of small molecule inhibitors of the TGFß and BMP pathways.. Cell Signal.

[40] Towner RA, Smith N, Saunders D, Brown CA, Cai X (2019). OKN-007 increases temozolomide (TMZ) sensitivity and suppresses TMZ-resistant glioblastoma (GBM) tumor growth.. Transl Oncol.

[41] Roy LO, Poirier MB, Fortin D (2018). Differential expression and clinical significance of transforming growth factor-β isoforms in GBM tumors.. Int J Mol Sci.

[42] Cao Z, Kyprianou N (2015). Mechanisms navigating the TGF-β pathway in prostate cancer.. Asian J Urol.

[43] Zhu B, Kyprianou N, Platanias LC (2005). Transforming growth factor β and prostate cancer.. Cytokines and cancer..

[44] Conery AR, Cao Y, Thompson EA, Townsend CM Jr, Ko TC (2004). Akt interacts directly with Smad3 to regulate the sensitivity to TGF-β induced apoptosis.. Nat Cell Biol.

[45] Akhurst RJ, Derynck R (2001). TGF-β signaling in cancer - a double-edged sword.. Trends in Cell Biology.

[46] Donkor MK, Sarkar A, Savage PA, Franklin RA, Johnson LK (2011). T cell surveillance of oncogene-induced prostate cancer is impeded by T cell-derived TGF-β1 cytokine.. Immunity.

[47] Jones E, Pu H, Kyprianou N (2009). Targeting TGF-β in prostate cancer: therapeutic possibilities during tumor progression.. Expert Opin Ther Targets.

[48] Yoshino A, Ogino A, Yachi K, Ohta T, Fukushima T (2010). Gene expression profiling predicts response to temozolomide in malignant gliomas.. Int J Oncol.

[49] Wang Z, Wang K, Wang R, Liu X (2017). SUMOylation regulates TGF-β1/Smad4 signalling in-resistant glioma cells.. Anticancer Drugs.

[50] Fruman DA, Meyers RE, Cantley LC (1998). Phosphoinositide kinases.. Annu Rev Biochem.

[51] Fresno Vara JA, Casado E, de Castro J, Cejas P, Belda-Iniesta C (2004). PI3K/Akt signalling pathway and cancer.. Cancer Treat Rev.

[52] Hunter T (2000). Signaling - 2000 and Beyond.. Cell.

[53] Gagliardi PA, Puliafito A, Primo L (2018). PDK1: at the crossroad of cancer signaling pathways.. Semin Cancer Biol.

[54] Haas B, Klinger V, Keksel C, Bonigut V, Kiefer D (2018). Inhibition of the PI3K but not the MEK/ERK pathway sensitizes human glioma cells to alkylating drugs.. Cancer Cell Int.

[55] Wen PY, Lee EQ, Reardon DA, Ligon KL, Alfred Yung WK (2012). Current clinical development of PI3K pathway inhibitors in glioblastoma.. Neuro Oncol.

[56] Hoesel B, Schmid JA (2013). The complexity of NF-κB signaling in inflammation and cancer.. Mol Cancer.

[57] Soubannier V, Stifani S (2017). NF-κB signalling in glioblastoma.. Biomedicines.

[58] Xia Y, Shen S, Verma IM (2014). NF-κB, an active player in human cancers.. Cancer Immunol Res.

[59] Bhat KLP, Balasubramaniyan V, Vaillant B, Ezhilarasan R, Hummelink K (2013). Mesenchymal differentiation mediated by NF-κB promotes radiation resistance in glioblastoma.. Cancer Cell.

[60] Rinkenbaugh AL, Cogswell PC, Calamini B, Dunn DE, Persson AI (2016). IKK/NF-κB signaling contributes to glioblastoma stem cell maintenance.. Oncotarget.

[61] Yu Z, Chen Y, Wang S, Li P, Zhou G (2018). Inhibition of NF-κB results in anti-glioma activity and reduces temozolomide-induced chemoresistance by down-regulating MGMT gene expression.. Cancer Letters.

[62] Cheng F, Guo D (2019). MET in glioma: signaling pathways and targeted therapies.. J Exp Clin Cancer Res.

[63] Huang M, Liu T, Ma P, Mitteer RA Jr, Zhang Z (2016). c-Met-mediated endothelial plasticity drives aberrant vascularization and chemoresistance in glioblastoma.. J Clin Invest.

[64] Li J, Li Q, Lin L, Wang R, Chen L (2018). Targeting the Notch1 oncogene by miR-139-5p inhibits glioma metastasis and epithelial-mesenchymal transition (EMT).. BMC Neurol.

[65] Bazzoni R, Bentivegna A (2019). Role of Notch signaling pathway in glioblastoma pathogenesis.. Cancers (Basel).

[66] Gersey Z, Osiason AD, Bloom L, Shah S, Thompson JW (2019). Therapeutic Targeting of the Notch Pathway in Glioblastoma Multiforme.. World Neurosurg.

[67] Venkatesh V, Nataraj R, Thangaraj GS, Karthikeyan M, Gnanasekaran A (2018). Targeting Notch signalling pathway of cancer stem cells.. Stem Cell Investig.

[68] Shih AH, Holland EC (2006). Notch signaling enhances nestin expression in gliomas.. Neoplasia.

[69] Hovinga KE, Shimizu F, Wang R, Panagiotakos G, Van Der Heijden M (2010). Inhibition of Notch signaling in glioblastoma targets cancer stem cells via an endothelial cell intermediate.. Stem Cells.

[70] Li WB, Ma MW, Dong LJ, Wang F, Chen LX (2011). MicroRNA-34a targets Notch1 and inhibits cell proliferation in glioblastoma multiforme.. Cancer Biol Ther.

[71] Hai L, Zhang C, Li T, Zhou X, Liu B (2018). Notch1 is a prognostic factor that is distinctly activated in the classical and proneural subtype of glioblastoma and that promotes glioma cell survival via the NF-κB(p65) pathway.. Cell Death Dis.

[72] Floyd DH, Kefas B, Seleverstov O, Mykhaylyk O, Dominguez C (2012). Alpha-secretase inhibition reduces human glioblastoma stem cell growth *in vitro* and *in vivo* by inhibiting Notch.. Neuro Oncol.

[73] Hellström M, Phng LK, Gerhardt H (2007). VEGF and Notch signaling: the yin and yang of angiogenic sprouting.. Cell Adh Migr.

[74] Zalles M, Smith N, Ziegler J, Saunders D, Remerowski S (2020). Optimized monoclonal antibody treatment against ELTD1 for GBM in a G55 xenograft mouse model.. J Cell Mol Med.

[75] Masiero M, Simões FC, Han HD, Snell C, Peterkin T (2013). A core human primary tumor angiogenesis signature identifies the endothelial orphan receptor ELTD1 as a key regulator of angiogenesis.. Cancer Cell.

[76] Zalles M, Smith N, Saunders D, Saran T, Thomas L (2020). Assessment of an scFv antibody fragment against ELTD1 in a G55 glioblastoma xenograft model.. Transl Oncol.

[77] Favara DM, Zois CE, Haider S, Pires E, Sheldon H (2019). ADGRL4/ELTD1 silencing in endothelial cells induces ACLY and SLC25A1 and alters the cellular metabolic profile.. Metabolites.

[78] Brennan CW, Verhaak RG, McKenna A, Campos B, Noushmehr H, TCGA Research Network (2013). The somatic genomic landscape of glioblastoma.. Cell.

[79] Libermann TA, Nusbaum HR, Razon N, Kris R, Lax I (1985). Amplification, enhanced expression and possible rearrangement of EGF receptor gene in primary human brain tumours of glial origin.. Nature.

[80] Fan QW, Cheng CK, Gustafson WC, Charron E, Zipper P (2013). EGFR phosphorylates tumor-derived EGFRvIII driving STAT3/5 and progression in glioblastoma.. Cancer Cell.

[81] Klingler S, Guo B, Yao J, Yan H, Zhang L (2015). Development of resistance to EGFR-targeted therapy in malignant gliomacan occur through EGFR-dependent and -independent mechanisms.. Cancer Res.

[82] Mellinghoff IK, Wang MY, Vivanco I, Haas-Kogan DA, Zhu S (2005). Molecular determinants of the response of glioblastomas to EGFR kinase inhibitors.. N Engl J Med.

[83] Reardon DA, Quinn JA, Vredenburgh JJ, Gururangan S, Friedman AH (2006). Phase 1 trial of gefitinib plus sirolimus in adults with recurrent malignant glioma.. Clin Cancer Res.

[84] Kreisl TN, Lassman AB, Mischel PS, Rosen N, Scher HI (2009). A pilot study of everolimus and gefitinib in the treatment of recurrent glioblastoma (GBM).. J Neurooncol.

[85] Hambardzumyan D, Gutmann DH, Kettenmann H (2016). The role of microglia and macrophages in glioma maintenance and progression.. Nat Neurosci.

[86] Schiffer D, Annovazzi L, Casalone C, Corona C, Mellai M (2018). Glioblastoma: microenvironment and niche concept.. Cancers (Basel).

[87] Parsa AT, Waldron JS, Panner A, Crane CA, Parney IF (2007). Loss of tumor suppressor PTEN function increases B7-H1 expression and immunoresistance in glioma.. Nat Med.

[88] Zhao W, Liu M, Kirkwood KL (2008). p38alpha stabilizes interleukin-6 mRNA via multiple AU-rich elements.. J Biol Chem.

[89] David CJ, Massagué J (2018). Contextual determinants of TGFβ action in development, immunity and cancer.. Nat Rev Mol Cell Biol.

[90] Spranger S, Bao R, Gajewski TF (2015). Melanoma-intrinsic β-catenin signalling prevents anti-tumour immunity.. Nature.

[91] Tomaszewski W, Sanchez-Perez L, Gajewski TF, Sampson JH (2019). Brain tumor microenvironment and host state: implications for immunotherapy.. Clin Cancer Res.

[92] Zhang X, Ding K, Wang J, Li X, Zhao P (2019). Chemoresistance caused by the microenvironment of glioblastoma and the corresponding solutions.. Biomed Pharmacother.

[93] Guan X, Hasan MN, Maniar S, Jia W, Sun D (2018). Reactive astrocytes in glioblastoma multiforme.. Mol Neurobiol.

[94] D’Alessio A, Proietti G, Sica G, Scicchitano BM (2019). Pathological and molecular features of glioblastoma and its peritumoral tissue.. Cancers (Basel).

[95] Placone AL, Quiñones-Hinojosa A, Searson PC (2016). The role of astrocytes in the progression of brain cancer: complicating the picture of the tumor microenvironment.. Tumour Biol.

[96] Munoz JL, Rodriguez-Cruz V, Greco SJ, Ramkissoon SH, Ligon KL (2014). Temozolomide resistance in glioblastoma cells occurs partly through epidermal growth factor receptor-mediated induction of connexin 43.. Cell Death Dis.

[97] Da Ros M, De Gregorio V, Iorio AL, Giunti L, Guidi M (2018). Glioblastoma chemoresistance: the double play by microenvironment and blood-brain barrier.. Int J Mol Sci.

[98] Barbero S, Bajetto A, Bonavia R, Porcile C, Piccioli P (2002). Expression of the chemokine receptor CXCR4 and its ligand stromal cell-derived factor 1 in human brain tumors and their involvement in glial proliferation in vitro.. Ann N Y Acad Sci.

[99] Sin WC, Aftab Q, Bechberger JF, Leung JH, Chen H (2016). Astrocytes promote glioma invasion via the gap junction protein connexin43.. Oncogene.

[100] Fioretti B, Castigli E, Micheli MR, Bova R, Sciaccaluga M (2006). Expression and modulation of the intermediate- conductance Ca^2+^-activated K^+^ channel in glioblastoma GL-15 cells.. Cell Physiol Biochem.

[101] Sciaccaluga M, Fioretti B, Catacuzzeno L, Pagani F, Bertollini C (2010). CXCL12-induced glioblastoma cell migration requires intermediate conductance Ca^2+^-activated K^+^ channel activity.. Am J Physiol Cell Physiol.

[102] Stegen B, Butz L, Klumpp L, Zips D, Dittmann K (2015). Ca^2+^-Activated IK K^+^ channel blockade radiosensitizes glioblastoma cells.. Mol Cancer Res.

[103] Morrone FB, Gehring MP, Nicoletti NF (2016). Calcium channels and associated receptors in malignant brain tumor therapy.. Mol Pharmacol.

[104] Costanza B, Umelo IA, Bellier J, Castronovo V, Turtoi A (2017). Stromal modulators of TGF-β in cancer.. J Clin Med.

[105] Coutinho de Souza P, Smith N, Atolagbe O, Ziegler J, Njoku C (2015). OKN-007 decreases free radical levels in a preclinical F98 rat glioma model.. Free Radic Biol Med.

